# Supplementary figures and images for: Enhancement of clinical signs in C3H/HeJ mice vaccinated with a highly immunogenic Leptospira methyl-accepting chemotaxis protein following challenge
Source: PLoS Negl Trop Dis. 2024 Sep 23;18(9):e0012155. doi: 10.1371/journal.pntd.0012155 (PMC11449317; doi:10.1371/journal.pntd.0012155)

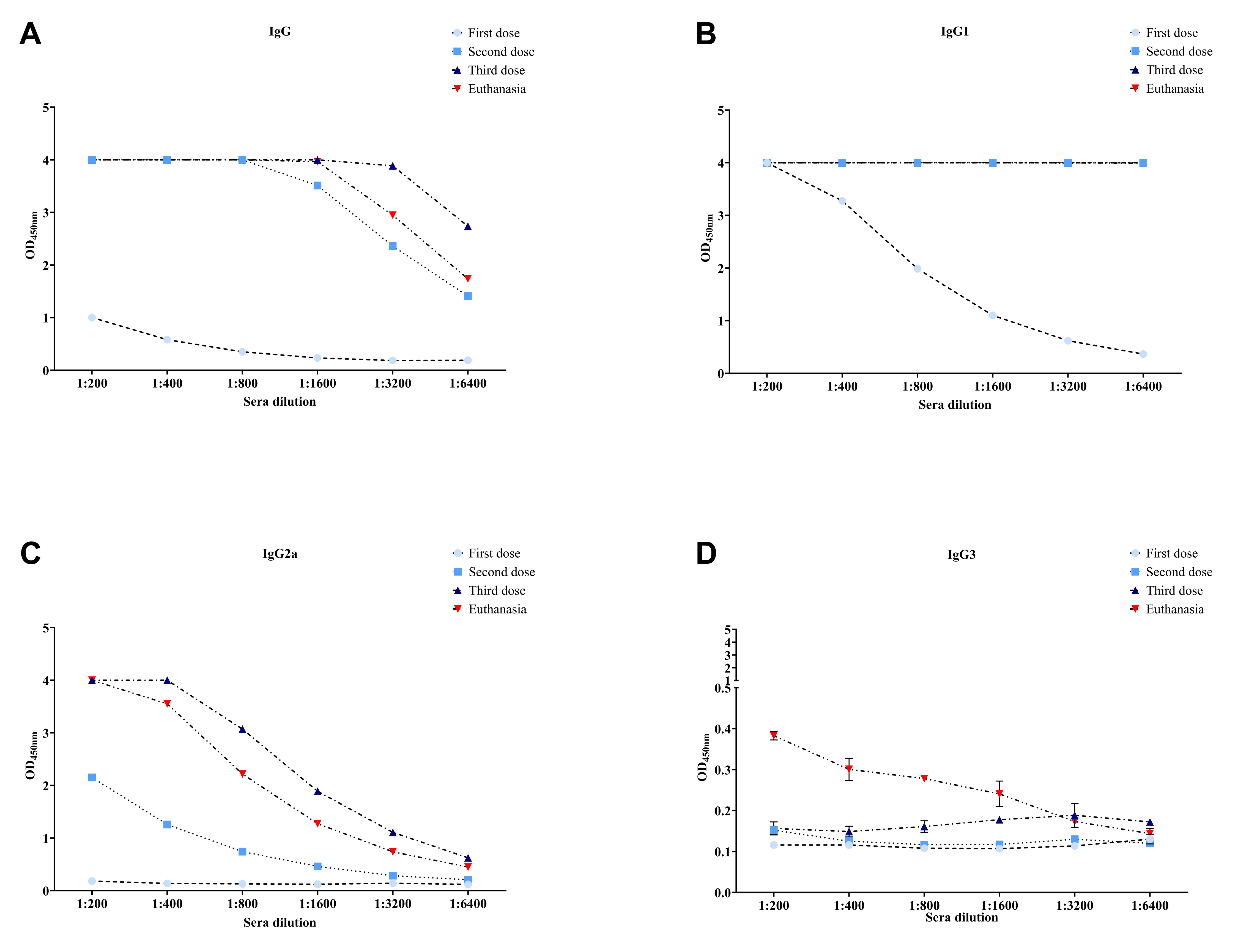

Supplement: S1 Fig — (A) Levels of IgG antibody. (B) Levels of IgG1 subclass. (C) Levels of IgG2a subclass. (D) Levels of IgG3 subclass. (OD) Optical density at 450 nanometers. The bars represent the standard error. All analyses were performed in technical triplicates. (TIF) [file pntd.0012155.s001.tif]

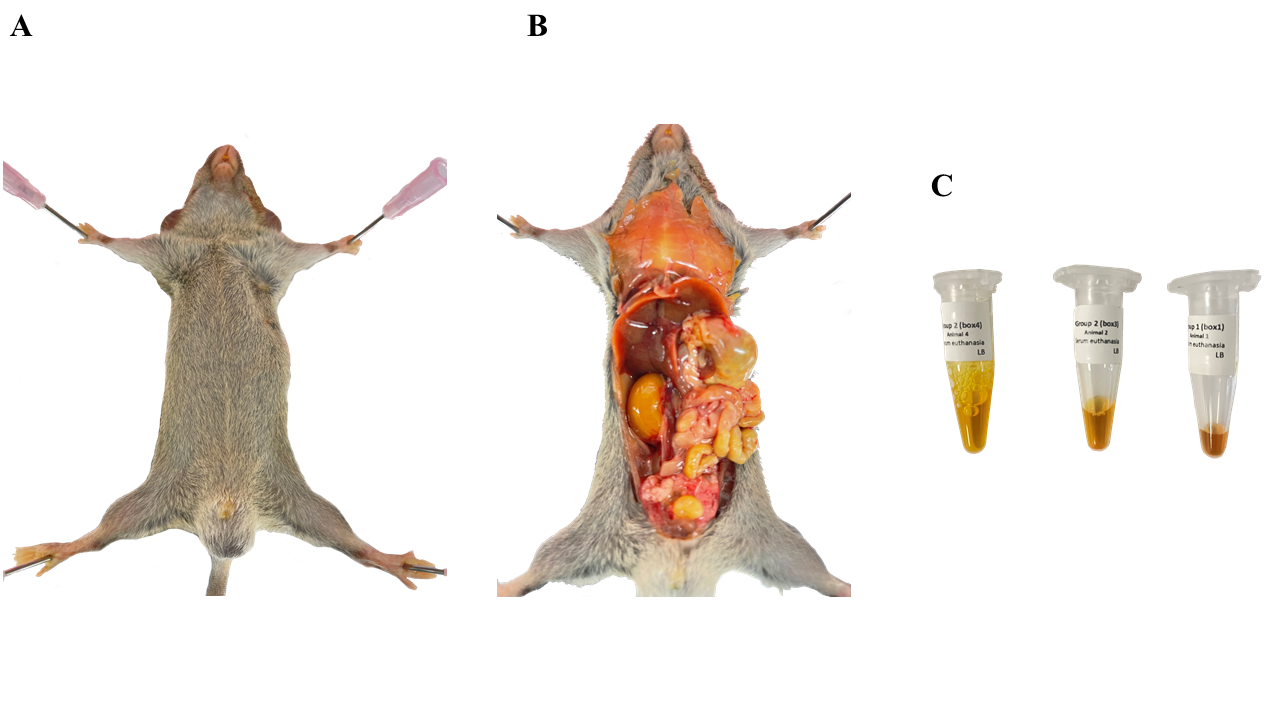

Supplement: S2 Fig — (A) External surface of body of infected mouse. (B) Internal organs of infected mice. (C) Sera samples collected at euthanasia of clinically ill animals. (TIF) [file pntd.0012155.s002.tif]
