# Supplementary material for: Enhancement of clinical signs in C3H/HeJ mice vaccinated with a highly immunogenic Leptospira methyl-accepting chemotaxis protein following challenge
Source: PLoS Negl Trop Dis. 2024 Sep 23;18(9):e0012155. doi: 10.1371/journal.pntd.0012155 (PMC11449317; doi:10.1371/journal.pntd.0012155)
Supplement: S1 Table — (DOCX) [file pntd.0012155.s003.docx]

**S1 Table. Clinical score to assess the condition of mice infected with pathogenic *Leptospira.***

| Clinical sign | Score | Action |
| --- | --- | --- |
| Normal behavior | 1 | None required |
| Well-conditioned (vertebrae and dorsal pelvis not prominent and palpable with slight pressure)^9^ |  |  |
| No loss of weight |  |  |
| Body temperature decreasing^1^ | 2 | Observe again in 2 hours  Increase the frequency of observation |
| Body weight decreasing (≤15% weight loss^2^) |  |  |
| Hair loss; Piloerection; Ruffled or spiked fur/rough coat; Lustreless/dry/scaled tail^2,5,6,7^ | 3 | Euthanize |
| Loss of motility; Hunched back^2,5,7^ |  |  |
| Decreased activity: Listlessness, Lethargy or sleep^4,5,6,8^ |  |  |
| Ocular and/or nasal discharge^5,7^ |  |  |
| Very restless^7^Ataxia or tremor^5,7^ |  |  |
| Cyanosis; Ears and paws presenting blue colour^5,7^ |  |  |
| Nasal bleeding^6^ |  |  |
| Jaundice^6^ |  |  |
| Isolation^7^ |  |  |
| Bulging or sunken eyes^7^ |  |  |
| Heavily soiled anal orifice^7^ |  |  |
| Hypothermia (> 4-6°C lost)^3^ |  |  |
| >20% weight loss^2^ |  |  |

References. (1) (1, 2); (2) (3, 4); (3) (5-7); (4) (7); (5) (8); (6) (9); (7) (10); (8) (11); (9) (12).

# **References**

1. Richer L, Potula HH, Melo R, Vieira A, Gomes-Solecki M. Mouse model for sublethal Leptospira interrogans infection. Infect Immun. 2015;83(12):4693-700.

2. Sullivan JP, Nair N, Potula HH, Gomes-Solecki M. Eyedrop Inoculation Causes Sublethal Leptospirosis in Mice. Infect Immun. 2017;85(4).

3. Nair N, Gomes-Solecki M. A Mouse Model of Sublethal Leptospirosis: Protocols for Infection with Leptospira Through Natural Transmission Routes, for Monitoring Clinical and Molecular Scores of Disease, and for Evaluation of the Host Immune Response. Curr Protoc Microbiol. 2020;59(1):e127.

4. Shetty A, Kundu S, Vernel-Pauillac F, Ratet G, Werts C, Gomes-Solecki M. Transient Presence of Live Leptospira interrogans in Murine Testes. Microbiol Spectr. 2022;10(3):e0277521.

5. Mei J, Riedel N, Grittner U, Endres M, Banneke S, Emmrich JV. Body temperature measurement in mice during acute illness: implantable temperature transponder versus surface infrared thermometry. Sci Rep. 2018;8(1):3526.

6. Stiles BG, Campbell YG, Castle RM, Grove SA. Correlation of temperature and toxicity in murine studies of staphylococcal enterotoxins and toxic shock syndrome toxin 1. Infect Immun. 1999;67(3):1521-5.

7. Olfert ED, Godson DL. Humane endpoints for infectious disease animal models. ILAR J. 2000;41(2):99-104.

8. Guidelines for the welfare of animals in rodent protection tests. A report from the Rodent Protection Test Working Party. Lab Anim. 1994;28(1):13-8.

9. Viriyakosol S, Matthias MA, Swancutt MA, Kirkland TN, Vinetz JM. Toll-like receptor 4 protects against lethal Leptospira interrogans serovar icterohaemorrhagiae infection and contributes to in vivo control of leptospiral burden. Infect Immun. 2006;74(2):887-95.

10. Beynen AC, Baumans V, Bertens AP, Havenaar R, Hesp AP, Van Zutphen LF. Assessment of discomfort in gallstone-bearing mice: a practical example of the problems encountered in an attempt to recognize discomfort in laboratory animals. Lab Anim. 1987;21(1):35-42.

11. Chaurasia R, Salovey A, Guo X, Desir G, Vinetz JM. Vaccination With Leptospira interrogans PF07598 Gene Family-Encoded Virulence Modifying Proteins Protects Mice From Severe Leptospirosis and Reduces Bacterial Load in the Liver and Kidney. Front Cell Infect Microbiol. 2022;12:926994.

12. Ullman-Cullere MH, Foltz CJ. Body condition scoring: a rapid and accurate method for assessing health status in mice. Lab Anim Sci. 1999;49(3):319-23.
