# Supplementary material for: Enhancement of clinical signs in C3H/HeJ mice vaccinated with a highly immunogenic Leptospira methyl-accepting chemotaxis protein following challenge
Source: PLoS Negl Trop Dis. 2024 Sep 23;18(9):e0012155. doi: 10.1371/journal.pntd.0012155 (PMC11449317; doi:10.1371/journal.pntd.0012155)
Supplement: S2 Table — (DOCX) [file pntd.0012155.s004.docx]

### S2 Table. Potential orthologs of MCP protein in 27 representative *Leptospira* genomes.

| **Species** | **Serovar** | **Strain** | **Gene ID^1^** | **Overall protein sequence identity (%)** | **Periplasmic domain sequence identity  (%)** | **BioProject^2^** |
| --- | --- | --- | --- | --- | --- | --- |
| *L. interrogans* | Canicola | Fiocruz LV133 | LEP1GSC069_2151 | 100 | 100 | PRJNA167235 |
| *L. interrogans* | N/A | FPW1039 | LEP1GSC079_4023 | 100 | 100 | PRJNA167242 |
| *L. interrogans* | Bataviae | L1111 | LEP1GSC087_3314 | 98 | 95 | PRJNA74089 |
| *L. interrogans* | Bratislava | PigK151 | BRAT_07470 | 98 | 95 | PRJNA269972 |
| *L. interrogans* | Bulgarica | Mallika | LEP1GSC007_2307 | 98 | 95 | PRJNA65041 |
| *L. interrogans* | Copenhageni | Fiocruz L1-130 | LIC_RS07205 | 98 | 95 | PRJNA224116 |
| *L. interrogans* | N/A | FPW2026 | LEP1GSC080_0670 | 98 | 96 | PRJNA74077 |
| *L. interrogans* | Grippothyphosa | UI 08368 | LEP1GSC097_1022 | 98 | 95 | PRJNA74101 |
| *L. interrogans* | Hardjo | Hardjoprajitno | LIH_11235 | 98 | 95 | PRJNA296687 |
| *L. interrogans* | Lai | 56601 | LA_2574 | 98 | 95 | PRJNA293 |
| *L. interrogans* | Lai | IPAV | LIF_A2104 | 98 | 95 | PRJNA32553 |
| *L. interrogans* | Linhai | 56609 | LIL_11527 | 98 | 95 | PRJNA217894 |
| *L. interrogans* | Manilae | UP-MMC-NIID LP | LIMLP_06865 | 98 | 94 | PRJNA287300 |
| *L. interrogans* | Muenchen | Brem 129 | LEP1GSC053_1863 | 98 | 95 | [PRJNA74063](https://www.ncbi.nlm.nih.gov/bioproject/PRJNA74063) |
| *L. interrogans* | Pomona | Pomona | LEP1GSC014_2450 | 98 | 95 | PRJNA65043 |
| *L. interrogans* | Pyrogenes | 2006006960 | LEP1GSC019_2521 | 98 | 95 | PRJNA74039 |
| *L. kirschneri* | N/A | H1 | LEP1GSC081_3597 | 95 | 90 | PRJNA74079 |
| *L. kirschneri* | N/A | H2 | LEP1GSC082_3188 | 95 | 90 | PRJNA167243 |
| *L. borgpetersenii* | Pomona | 200901868 | LEP1GSC133_2538 | 72 | 62 | PRJNA167255 |
| *L. weilii* | N/A | UI 13098 | LEP1GSC108_2188 | 71 | 63 | PRJNA74123 |
| *L. mayottensis* | N/A | 200901116 | LEP1GSC190_11120 | 71 | 64 | PRJNA167259 |
| *L. borgpetersenii* | Hardjo | JB197 | LBJ_RS05970 | 70 | 63 | PRJNA224116 |
| *L. borgpetersenii* | Hardjo | L550 | LBL_RS06390 | 70 | 63 | PRJNA224116 |
| *L. santarosai* | Shermani | LT 821 | LSS_11685 | 70 | 62 | PRJNA47139 |
| *L. licerasiae* | Varillal | VAR 010 | LEP1GSC185_2696 | 44 | 23 | PRJNA74167 |
| *L. biflexa* | Patoc | Patoc 1 (Ames) | LBF_RS17995 | 31 | 23 | PRJNA224116 |
| *L. biflexa* | Patoc | Patoc 1 (Paris) | LEPBI_RS17980 | 31 | 23 | PRJNA224116 |

^1^Locus tag of the corresponding entry in the annotated genome for the specimen.

^2^Accession number of the NCBI BioProject providing access to the annotated genome on GenBank.

Periplasmic domain: residues 217 to 457.

(%) Percentage.

(N/A) Not available.
