# Supplementary material for: Enhancement of clinical signs in C3H/HeJ mice vaccinated with a highly immunogenic Leptospira methyl-accepting chemotaxis protein following challenge
Source: PLoS Negl Trop Dis. 2024 Sep 23;18(9):e0012155. doi: 10.1371/journal.pntd.0012155 (PMC11449317; doi:10.1371/journal.pntd.0012155)
Supplement: S3 Table — (DOCX) [file pntd.0012155.s005.docx]

**S3 Table. MCP protein information obtained from NCBI and microarray used for selection of vaccine antigen.**

| **Protein identification** | **Predicted protein classification** | **Organism** | **Full-length protein sequence** | **Original reactive peptide** | **Reactive epitope of the peptide** | **Number of amino acids** | **Molecular weight of recombinant fragment (kDa)** |
| --- | --- | --- | --- | --- | --- | --- | --- |
| LEP1GSC069_2151 | Methyl-accepting chemotaxis protein signaling domain protein | *Leptospira interrogans* serovar Canicola str. Fiocruz LV133 | MNDNITNSIRKFILHFILVTEVVGFTLTIGIAIVFFTTFLEMDSDQLKIAIRITLTTAVFTLMFAIFSDTCRLRPIHKYLFMLEKGITDKQIALNAQKSIFRIPFFHSIDIGLRILVTAFVVIYLLSQFIILETADYYNLGSLTLIMCLLVGVYTFFASEQLTFNLIKSGVFDHINISSLTKVRLTRSLTITFIFIVFVLAITVSGLVFKLNYSGIRKSYFNQMNNMNETLSIFAESIFEEVRSDSEKLKSDPFFISLIKNYKKDEIQNFLKTLLERSPIYESISLIKPENQSWKVIAGTETLSQKTDSILKDFQLPSENVVLETISKHKTFFIKPISSPISETPVLLILETIFENPNLFIAYSLKITDLTQKIIGSIQIGKSGHIGFMDREETVINHINSSLYLKKLKNIPFYKQIKNYNYDVPIRFLSNGKYRYIIFHKNKKYNFITFTSIENEEIAGEAIICVYVMTGISFFGLSFIGILIYLILRKRIRPLEESRKVLESMTGGDLTKGLQVFSMDEIGEMSVSINLFNKKIKKILNKIITASENLAGSSDEMSRALNFISANTQNQAASSEEISSSIEEIVAGMNGVEIQTNEQVSLLNQLASDMNQFSDSIHKISHNMEKTMSEVERITEEAKKGGNSLELT***NHSITKISN*SSEDISGV**IEIINTISEQIHLLALNAAIEAARAGNAGRGFAVVADEISKLADKTTNSIKDIERIIKNNETEIGAGIQNITDTVNVITGIIEGISEINHQMKVVNQFMENQLSKNDQMNLTTKEVKDRADVIQISVQEQKNAIEEISKTTATINELNQSSAASSEELSSNSIGLAKLAEDLKHEVVFFKL | NHSITKISNSSEDISGV | NHSITKISN | 198  (649-846) | 22 |

| (kDa) Molecular weight measured in kilodaltons.  (Bold) Original peptide sequence containing 17 amino acids. | |
| --- | --- |
| (Bold, italic and underlined) Reactive epitope in the peptide. |  |
