# Supplementary material for: Enhancement of clinical signs in C3H/HeJ mice vaccinated with a highly immunogenic Leptospira methyl-accepting chemotaxis protein following challenge
Source: PLoS Negl Trop Dis. 2024 Sep 23;18(9):e0012155. doi: 10.1371/journal.pntd.0012155 (PMC11449317; doi:10.1371/journal.pntd.0012155)
Supplement: S4 Table — (DOCX) [file pntd.0012155.s006.docx]

| **Group identification**  **S4 Table. Summary of results from individual mouse from the MCP-vaccinated and PBS+Alhydrogel** **control groups.**  **Ct** | **Animal** | **Days to Euthanasia** | **Jaundice** | **Histopathology changes in kidney** | **qPCR results** **Ct values** | | | | | **Culture** |
| --- | --- | --- | --- | --- | --- | --- | --- | --- | --- | --- |
|  |  |  |  |  | **Kidney** | **Lungs** | **Spleen** | **Heart** | **Liver** | **Kidney** |
| MCP vaccine | 1 | 7 | Moderate | Hyaline casts: mild Lymphoplasmacytic interstitial nephritis: minimal Tubular cytoplasmic blebbing | Positive  27.027 | Positive 29.68 | Positive 29.389 | Positive 29.039 | Positive 26.19 | Positive |
|  | 2 | 7 | Severe | Hyaline casts: minimal  Tubular cytoplasmic blebbing  Tubular degeneration and necrosis: mild to moderate | Positive  26.673 | Positive 29.542 | Positive 34.365 | Positive 36.566 | Positive 32.159 | Positive |
|  | 3 | 6 | Severe | Hyaline cast: mild  Tubular degeneration and necrosis: minimal to mild | Positive  27.984 | Positive 27.906 | Positive 30.029 | Positive 33.556 | Positive 26.112 | Positive |
|  | 4 | 28 | - | Periglomerular fibrosis | Positive  18.774 | Positive 29.691 | Positive 31.038 | Positive 28.016 | Positive 32.385 | Positive |
|  | 5 | 7 | Moderate | Hyaline casts: mild  Tubular cytoplasmic blebbing  Tubular degeneration and necrosis: minimal to mild  Tubular dilation | Positive 28.6845 | Positive 28.375 | Positive 31.447 | Positive 34.338 | Positive 25.928 | Positive |
|  | 6 | 10 | - | Hyaline casts: mild  Lymphoplasmacytic interstitial nephritis: mild to moderate  Lymphoplasmacytic pyelitis and neutrophilic tubulointerstitial nephritis  Tubular cytoplasmic blebbing  Tubular dilation | Positive 26.7505 | Positive 30.288 | Positive 34.784 | Positive 37.719 | Positive 35.01 | Negative |
|  | 7 | 6 | Severe | Hyaline casts: mild  Proximal tubular degeneration: mild  Tubular cytoplasmic blebbing | Positive 25.883 | Positive 27.809 | Positive 29.736 | Positive 34.801 | Positive 24.162 | Negative |
|  | 8 | 5 | - | Acute papillary necrosis  Proximal tubular degeneration: mild  Tubular mineralization: moderate | Positive 24.522 | Positive 24.903 | Positive 24.771 | Positive 29.097 | Positive 22.538 | Negative |
| **Group identification** | **Animal** | **Days to Euthanasia** | **Jaundice** | **Histopathology changes in kidney** | **qPCR results** **Ct values** | | | | | **Culture** |
|  |  |  |  |  | **Kidney** | **Lungs** | **Spleen** | **Heart** | **Liver** | **Kidney** |
| PBS+Alhydrogel | 1 | 10 | - | Lymphoplasmacytic tubulointerstitial nephritis: mild  Tubular degeneration and necrosis with atrophy and fibrosis: moderate to marked  Tubular regeneration  Tubular mineralization: rare | Positive 25.2805 | Positive 32.431 | Positive 34.703 | Positive 38.797 | Positive 34.449 | Positive |
|  | 2 | 7 | Moderate | Acute tubular degeneration and necrosis: mild  Hyaline casts: moderate | Positive 29.2855 | Positive 27.324 | Positive 31.937 | Positive 30.06 | Positive 24.098 | Negative |
|  | 3 | 10 | - | Hyaline casts: minimal to mild  Lymphoplasmacytic interstitial nephritis: minimal to moderate  Lymphoplasmacytic pyelitis  Tubular degeneration, necrosis and atrophy: marked  Tubular regeneration | Positive 26.985 | Positive 32.241 | Positive 33.194 | Negative | Positive 32.719 | Positive |
|  | 4 | 10 | - | Lymphoplasmacytic tubulointerstitial nephritis: minimal to moderate  Tubular degeneration, necrosis with atrophy and fibrosis: marked  Tubular regeneration  Tubular mineralization: minimal to mild | Positive 26.6935 | Positive 33.895 | Positive 33.243 | Positive 37.257 | Positive 36.312 | Positive |
|  | 5 | 28 | - | Lymphoplasmacytic and neutrophilic Pyelitis: mild to moderate  Lymphoplasmacytic interstitial nephritis: mild radiating  Tubular degeneration, necrosis and atrophy: mild to moderate  Tubular atrophy: mild | Positive 22.8325 | Positive 27.647 | Positive 33.191 | Positive 29.624 | Positive 30.652 | Positive |
|  | 6 | 28 | - | Lymphoplasmacytic interstitial nephritis: minimal to mild  Periglomerular fibrosis: minimal to mild  Tubular hyaline casts: mild  Tubular degeneration, necrosis, and atrophy: minimal to mild | Positive 20.435 | Positive 30.782 | Positive 28.893 | Positive 33.825 | Positive 29.326 | Positive |
|  | 7 | 28 | - | Lymphoplasmacytic interstitial nephritis: mild  Lymphoplasmacytic and neutrophilic pyelitis: mild to moderate  Periglomerular fibrosis  Tubular degeneration, necrosis and atrophy with interstitial fibrosis: mild | Positive 20.338 | Positive 25.219 | Positive 32.346 | Positive 29.453 | Positive 27.859 | Positive |
|  | 8 | 7 | Severe | Interstitial nephritis: minimal  Periglomerular fibrosis  Proximal tubular ectasia with cytoplasmic blebbing: moderate  Tubular degeneration, necrosis, and atrophy with mild interstitial fibrosis: moderate to marked  Tubular hyaline casts and mineralization: mild | Positive 30.1125 | Positive 30.991 | Positive 32.177 | Positive 31.9 | Positive 25.637 | Negative |

(Ct) Threshold cycles.

**Ct**
